# *Staphylococcus aureus* increases Prostaglandin E_2_ secretion in cow neutrophils by activating TLR2, TLR4, and NLRP3 inflammasome signaling pathways

**DOI:** 10.3389/fmicb.2023.1163261

**Published:** 2023-04-24

**Authors:** Kai Zhang, Yan Jia, Yinghong Qian, Xueying Jiang, Shuangyi Zhang, Bo Liu, Jinshan Cao, Yongli Song, Wei Mao

**Affiliations:** ^1^College of Veterinary Medicine, Inner Mongolia Agricultural University, Huhhot, China; ^2^Key Laboratory of Animal Clinical Treatment Technology, Ministry of Agriculture, Huhhot, China; ^3^Inner Mongolia Academy of Agricultural and Animal Husbandry Sciences, Huhhot, China; ^4^Stem Cell and Microbiology, Inner Mongolia University, Huhhot, China

**Keywords:** *Staphylococcus aureus*, Prostaglandin E_2_, neutrophils, toll-like receptor 2, toll-like receptor 4, NLR pyrin domain-containing 3

## Abstract

**Introduction:**

In clinical settings, dairy cows are often attacked by pathogenic bacteria after delivery, especially *Staphylococcus aureus* (*S. aureus*). Neutrophils have long been regarded as essential for host defense against *S. aureus*. Prostaglandin E_2_ (PGE_2_) can additionally be used as an inflammatory mediator in pathological conditions to promote the repair of inflammatory injuries. However, whether *S. aureus* can promote the accumulation of PGE_2_ after the infection of neutrophils in cows and its mechanism remain unclear. Lipoprotein is an important immune bioactive ingredient of *S. aureus*.

**Methods:**

In this study, the changes in neutrophils were monitored in dairy cows infected with wild-type *S. aureus* (SA113) and an *S. aureus* lipoprotein-deficient strain (Δ*lgt*); meanwhile, we established whether pattern recognition receptors mediate this process and whether *S. aureus* lipoproteins are necessary for causing the release of PGE_2_ from cow neutrophils.

**Results:**

The results showed that Δ*lgt* was less effective than SA113 in inducing the production of IL-1β, IL-6, IL-8, IL-10, and PGE_2_ within neutrophils; furthermore, TLR2, TLR4, and NLRP3 receptors were found to mediate the inducible effect of lipoprotein on the above inflammation mediators and cytokines, which depended on MAPK and Caspase-1 signaling pathways. In addition, TLR2, TLR4, and NLRP3 inhibitors significantly inhibited PGE_2_ and cytokine secretion, and PGE_2_ was involved in the interaction of *S. aureus* and neutrophils in dairy cows, which could be regulated by TLR2, TLR4, and NLRP3 receptors. We also found that *S. aureus* was more likely to be killed by neutrophils when it lacked lipoprotein and TLR2, TLR4, and NLRP3 were involved, but PGE_2_ seemed to have no effect.

**Discussion:**

Taken together, these results suggest that lipoprotein is a crucial component of *S. aureus* in inducing cytokine secretion by neutrophils as well as killing within neutrophils, which could be accomplished by the accumulation of PGE_2_ by activating MAPK and the Caspase-1 signaling pathways through TLR2, TLR4, and NLRP3 receptors. These results will contribute to a better understanding of the interaction between *S. aureus* and host immune cells in dairy cows.

## Introduction

In veterinary clinical practice, *Staphylococcus aureus*(*S. aureus*) is one of the main bacteria that cause infectious diseases in dairy cows, such as mastitis, vaginitis (Petit et al., 2010), and endometritis (Zhao et al., 2014). Neutrophils play a role in innate immunity and are vital for the clearance of *S. aureus* (Dsa et al., 2022). Unlike other phagocytes such as monocytes and macrophages, neutrophils are fully capable of killing gram-positive bacteria such as *S. aureus* (Kevin and DeLeo, 2012). Therefore, neutrophils are the most prominent immune cells, which can effectively kill *S. aureus* in the immune system (Amulic et al., 2012). Prostaglandin E_2_ (PGE_2_) performs an indispensable function in endometrial inflammatory harm (Tingting et al., 2018). It builds up in pathogen-infected endometrial tissues and increases the expression of pro-inflammatory proteins and damage-associated molecular patterns (DAMPs), which increased the degree of the inflammatory response (Tingting et al., 2018). However, the mechanisms underlying the role of PGE_2_ accumulation in *S. aureus*-infected cow neutrophils remain unclear.

The innate immune system plays a crucial role in the host's defense against infections brought on by *S. aureus*, a well-known gram-positive bacterium. Notably, many virulence elements of *S. aureus* are directed toward factors of the innate immune system (Kessel et al., 2014). Toll-like receptor 2 (TLR2) is a class I transmembrane receptor that recognizes lipophosphatidic acid (LTA) and peptidoglycan (PGN) of *S. aureus*, thereby mediating the innate immune inflammatory response (Nakanishi et al., 2008). *S. aureus* lipoproteins are ligands for the major TLR2 receptor in cell wall components (Hashimoto et al., 2006a,b). The transcription factors such as mitogen-activated protein kinase (MAPK) signaling pathways are activated when TLR2 receptors are stimulated by *S. aureus*, which promotes the secretion of many pro-inflammatory mediators (Sheldon et al., 2014; Tingting et al., 2019). It was found that neutrophils express TLR2 and Toll-like receptor 4 (TLR4), and brain hemorrhage is not only dependent on TLR2 receptors but also requires TLR4 receptors. Increased expression of TLR2 and TLR4 receptors is linked to functional deficits within 3 months of a brain hemorrhage. TLR4 receptors are the first TLRs identified in mammals that are homologous to Drosophila and are the main receptors recognized by lipopolysaccharide (LPS), a cell wall component of gram-negative bacteria (Rodríguez-Yáñez et al., 2012). TLR2 and TLR4 are the two widely studied receptors that regulate the phagocytic killing of gram-positive and gram-negative bacteria by polymorphonuclear neutrophils (PMNs), respectively (Prince et al., 2011). It is believed that the IL-1β produced by the NLRP3-inflammasome activates Caspase-1, which causes cyclooxygenase-2 (COX-2) to be upregulated and PGE_2_ to be produced more often (Catley et al., 2003). It was demonstrated that not only TLR2 is involved in the defense of *S. aureus* infection in mouse peritoneal macrophages but also TLR4 and the NLR pyrin domain-containing 3 (NLRP3) inflammasome can be activated by *S. aureus* (Wu et al., 2020). However, it is not clear how cow neutrophils recognize *S. aureus*.

Among the PG_S_, PGE_2_ is mostly related to inflammation and is acknowledged to be present in large amounts in inflammatory exudates. In particular, PGE_2_ is present in higher concentrations during inflammation compared to other PG_S_, and it is involved in all responses leading to the typical symptoms of inflammation, including fever, edema, and pain (Wan et al., 2018). PGE_2_ production and biosynthesis, through inflammation, are exceptionally regulated by COX-2 and microsomal PGE synthase (mPGES)-1 and are induced by a variety of pro-inflammatory factors, which include interleukin-6 (IL-6), tumor necrosis factor-α (TNF-α), and interleukin-1β (IL-1β) (Pecchi et al., 2009). Preliminary laboratory discovery shows that the use of TLR2, TLR4, and NLRP3-deficient mice in tests revealed that these proteins are involved in macrophage PGE_2_ secretion in response to *S. aureus* (Wu et al., 2020). However, it is still unclear whether PGE_2_ accumulation in cow neutrophils infected with *S. aureus* is regulated by TLR2, TLR4, or NLRP3 receptors.

In this study, we found how *S. aureus* lipoprotein and host inflammatory bodies of TLR2, TLR4, and NLRP3 affected neutrophils' ability to secrete PGE_2_ in cows with *S. aureus* infections.

## Materials and methods

### Reagents, chemicals, and antibodies

Fetal bovine serum (FBS), RPMI1640 medium, and PBS (Hyclon, Logen, UT); dairy cow peripheral blood neutrophil isolation kit (Hao Yang, Tian Jing, China); MH broth (Oxoid, Hampshire, UK); TLR2 receptor inhibitor (C29, MCE, China), TLR4 receptor inhibitor (TAK-242, MCE, China), NLRP3 receptor inhibitor (MCC950, MCE, China); COX-2 inhibitor (CAY10404), mPGES-1 inhibitor (MF63) (Cayman Chemical, MI, United States); gentamicin sulfate(Promega, Wisconsin, United States); Bovine IL-1β, IL-10, IL-8 ELISA Kits (Kingfisher, Biotech); Bovine IL-6 ELISA Kit (R&D Systems, California, United States); PGE_2_ ELISA Kit (Cayman Chemical, MI, United States); M-PER mammalian protein extraction reagent, Halt protease inhibitor, pre-stained protein ladders, blocking buffer, starting block T20 (TBS), LIVE/DEAD Bac Light Bacterial Viability Kits (Thermo Fisher Scientific); SDS-PAGE loading buffer (Takara, Shiga, Japan); Pierce BCA Protein Assay Kit (Rockford, United States); SDS-PAGE gel electrophoresis kit (Solarbio, Beijing, China); Axy Prep Multisource Total mRNA Miniprep Kit (Axygen Scientific, Union City, United States); Primer Script RT Master Mix (Takara); SYBR Green Master (Rox) (Roche, Basel, Switzerland). All primers were synthesized by Generay (Shanghai, China).

### Bacterial strains and animals

Prof. Friedrich Götz (Mikrobielle Genetik, Universität Tübingen, Germany) kindly contributed to the *S. aureus* SA113 wild-type strain (WT; ATCC 35558) and an *S. aureus* SA113 isogenic mutant lgt::ermB (Δ*lgt*) deficient in lipoprotein maturation (Stoll et al., 2005). At a constant temperature of 37°C for 16 h, all bacterial strains were grown in MH broth to an optical density of 2.0 at 600 nm. The 4~6-year-old Holstein cows were provided by the Veterinary College of Inner Mongolia Agricultural University.

### Isolation of peripheral blood neutrophils

Jugular vein blood was collected from healthy Holstein cows. The cells were isolated using a bovine peripheral blood neutrophil extraction kit. A measure of 4 mL of separation solution I from the kit was added to a 15-mL centrifuge tube, and then 3 mL of the fresh anticoagulant was slowly added. The solution was centrifuged horizontally for 25 min at 2,000 rpm/min. The cells in the white layer at the interface of the separation solution were collected and put into a 50 mL centrifuge tube. In total, 2 mL of separation fluid II was increased to the new 15 mL centrifuge tube, and the collected white layer cells were slowly added to it. It was centrifuged horizontally at 2,000 rpm/min for 40 min. The supernatant was thrown out, the cells were collected, and 2 ml of RBC lysate was added for 2 min before being neutralized with a PBS solution three times the volume and centrifuged for 5 min at 800 g. Red blood cells that were still present were cleaved once more and then re-suspended in RPMI 1640 medium with 10% FBS before being counted and put in a 6-well plate (Ciliberti et al., 2021).

### Experimental treatments

Dairy cow neutrophils were placed 2 × 10^6^ cells per well on 6-well culture plates at 37°C in a 5% CO_2_ cell culture incubator. Two strains of *S. aureus* SA113 and Δ*lgt* were, respectively, infected with cells at 6 × 10^6^ (MOI 3:1) and 2 × 10^7^ (MOI 10:1). The infected cells were cultivated for 1 h at 37°C in an incubator, and gentamicin sulfate with a final concentration of 100 μg/ml was added to kill the extracellular *S. aureus*. In the TLR2 (C29, 10^−5^ M), TLR4 (TAK-242, 10^−5^ M), and NLRP3 (MCC950, 10^−5^ M) inhibitor groups, TLR2, TLR4, and NLRP3 inhibitors were added 4 h before *S. aureus* addition. In the COX2 (CAY10404, 10^−5^ M) and mPGES-1 (MF63, 10^−5^ M) inhibitor groups, the inhibitor was added 1 h in advance.

### ELISA

Different cytokines were separately tested according to the instructions provided with each ELISA assay kit, the corresponding standard curves were plotted, and the cytokine samples' concentrations were calculated. A total of three biological replicates were performed.

### Western blot analysis

Cells were treated with an M-PER reagent, and total protein extraction was carried out on the ice. According to the manufacturer's instructions, concentration measurements and protein denaturation were carried out. A measure of 20 μg of full protein on each lane was divided by SDS-PAGE (12%) electrophoresis at 80 V, and the protein was transferred to polyvinylidene fluoride membranes at 25 V for 30 min. The membranes were blocked with TBST containing 3% BSA for 4 h at room temperature. The primary antibody is diluted to the appropriate concentration and incubated at 4°C for 14 h the dilution ratio of primary antibody is shown in Table 1. The membranes were washed five times with TBST for 5 min, each after incubation. The secondary antibody was incubated at room temperature for 1 h (1:3,000). The membranes were, then, subjected to three 20 min TBST washes after incubation, and after that exposed to electrochemiluminescence film and Western blot detection tools. Using ImageJ, band densities were measured.

**Table 1:** List of major antibodies.

**Antibody name**	**Dilution ratio**	**Producer**
COX-2	1:1,000	Abcam, Cambridge, UK
mPGES-1	1:1,000	Cayman Chemical, MI, USA
Phospho-ERK	1:1,000	Cell Signaling Technology,
ERK	1:1,000	Beverly, MA
Phospho-p38	1:1,000	Ditto
p38	1:1,000	Ditto
Phospho-JNK	1:1,000	Ditto
JNK	1:1,000	Ditto
β-actin	1:1,000	Ditto
Pro-caspase-1 + p10 + p12	1:1,000	Abcam, Cambridge,
TLR2, TLR4	1:200	UKNovus Biologicals, USA
NLRP3	1:200	Novus Biologicals, USA

### Real-time RT-PCR analysis

After *S. aureus*-infected neutrophils, total RNA was isolated. Total mRNA extraction and reverse transcription were performed. The polymerase chain reaction conditions were as follows: 50°C, 2 min; 95°C, 10 min; 95°C, 15 s; and 60°C, 60 s, for 40 cycles. The annealing temperature was 58°C. The primers used in this study are listed in Table 2. The results were calculated using the 2^−ΔΔCt^ calculation method.

**Table 2:** Primer sequences for RT-qPCR.

**Gene name**	**Sequences(5^′^-3^′^)**	**Accession number**
*β-actin*	F:5′-CCAAGGCCAACCGTGAGAAGAT-3′ R:5′-CCACGTTCCGTGAGGATCTTCA-3′	NM_173979.3
*TLR2*	F:5′-ATGATGCTGCCATTCTGATTCT-3′ R:5′-CTCCAGGTAGGTCTTGGTGTTC-3′	NM_174197.2
*TLR4*	F:5′-AGGTAGCCCAGACAGCATTTC-3′ R:5′-AGCGAGTGGAGTGGTTCATAA-3′	NM_174198.6
*NLRP3*	F:5′-TCCCTGACCAGACTCTACTTG-3′ R:5′-GTGGGTGAGATTCTGATTTGTA-3′	NM_001102219.1
*COX-2*	F: 5′-GGTGCCTGGTCTGATGATGT-3′ R:5′-GATTAGCCTGCTTGTCTGGAAC-3′	XM-007115297.3
*mPGES-1*	F: 5′-ATGGTACACACCGTGGCATA-3′ R: 5′-CACAATCTCAAAGGGCCATC-3′	XM-027556544.1

### Microscopical examination of neutrophil cytotoxicity to bacteria

To verify the effect of PGE2, TLR2, TLR4, and NLRP3 on phagocytosis, neutrophils were cultured in 35 mm glass-bottomed Petri dishes (1 × 10^5^ cells/dish). Cells were pretreated, or not treated, with a selective mPGES-1 inhibitor MF63, a TLR2 receptor inhibitor C29, a TLR4 receptor inhibitor TAK-242, and an NLRP3 receptor inhibitor MCC950. The cell membrane is, then, labeled with SYTO9. *S. aureus* SA113 or Δ*lgt* strain (MOI 30:1) was labeled with PI for 6 h and then fixed with 4% paraformaldehyde. Laser confocal microscopy is used to capture images (400 X). Images of different samples were collected under the same conditions.

### Statistical analysis

All data had been analyzed with the use of GraphPad Prism 5 and are expressed as the mean ± standard deviation (SD). Statistical significance was evaluated by the one-way analysis of variance (ANOVA) followed by Tukey's multiple comparison test or two-way ANOVA (Bonferroni's post-test), as appropriate. Differences with *P* ≤ 0.05 were considered statistically significant (^*^P < 0.05; ^**^P < 0.01; and ^***^P < 0.001).

## Results

### *Staphylococcus aureus* lipoprotein is an important protein that can stimulate the secretion of PGE_2_ and cytokines by dairy cow neutrophils, as well as activate TLR2, TLR4, and NLRP3 receptors and the MAPK and Caspase-1 signaling pathways

Whether lipoprotein can induce the secretion of PGE_2_ and cytokines after the infection of neutrophils by *S. aureus* is unclear. Moreover, the activation of TLR2, TLR4, and NLRP3 receptors and their mediated MAPK and Caspase-1 signaling pathways are still unclear. To analyze the effects of *S. aureus* on the secretion of PGE_2_ and cytokines in neutrophils, the activation of TLR2, TLR4, and NLRP3 receptors, and the mediated MAPK and Caspase-1 signaling pathways, ELISA was used to detect the secretion of PGE_2_, IL-1β, IL-10, IL-6, and IL-8. The phosphorylation levels of ERK, JNK, P38, and Caspase-1 were detected by Western blotting. TLR2, TLR4, and NLRP3 receptors' mRNA levels were detected by qRT-PCR and Western blotting.

**Figure 1:**
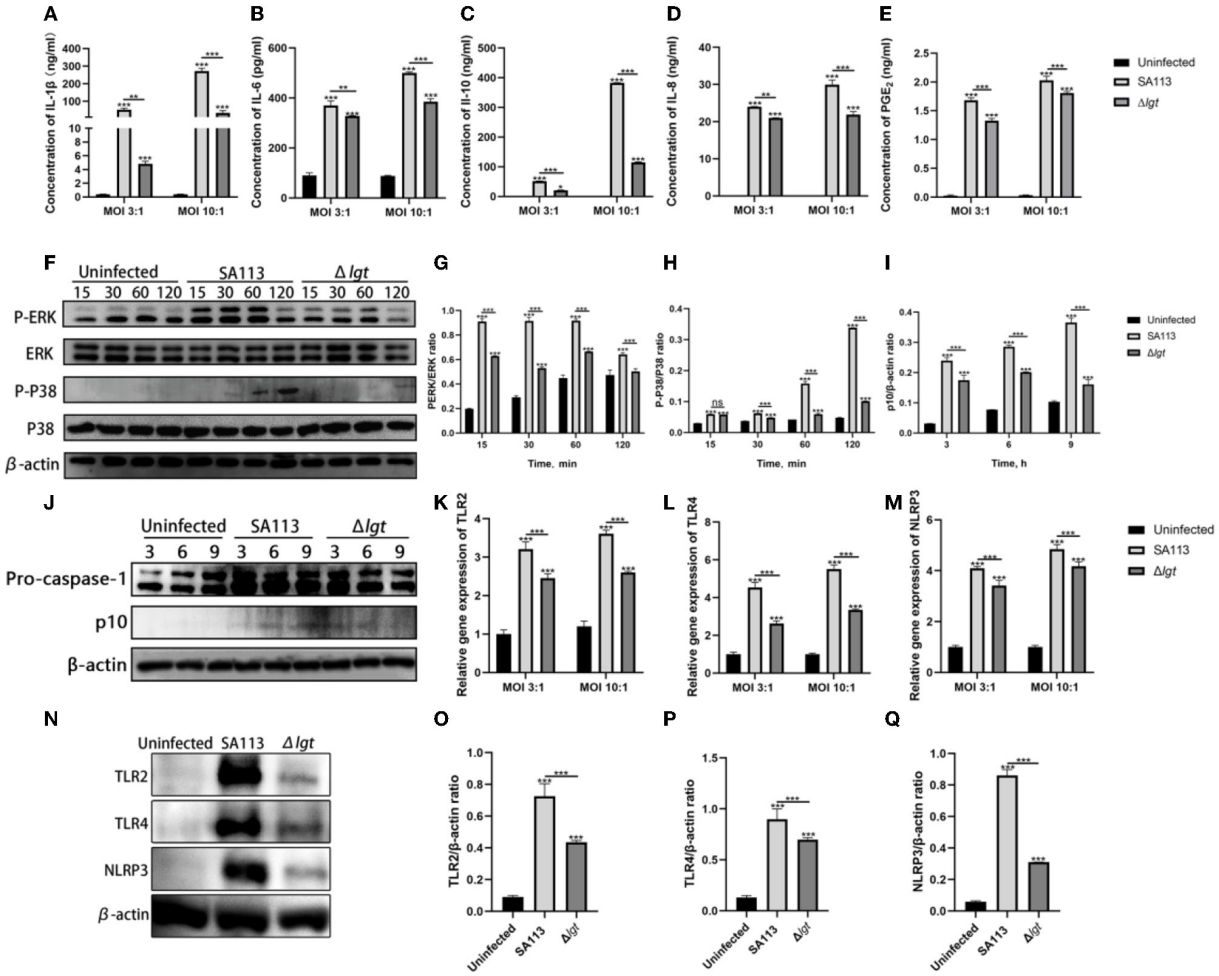
*Staphylococcus aureus* lipoprotein is an important protein that can induce the secretion of PGE_2_ and cytokines by neutrophils in dairy cows and can also activate TLR2, TLR4, and NLRP3 receptors and their mediated MAPK and Caspase-1 signaling pathways. ELISA results showed the secretion levels of cytokines IL-1β **(A)**, IL-6 **(B)**, IL-10 **(C)**, IL-8 **(D)**, and PGE_2_ **(E)** in the uninfected, SA113, and Δ*lgt* groups after neutrophil infection with MOI 3:1 and MOI 10:1. Western blot analysis revealed the activation of the MAPK **(F–H)** and Caspase-1 **(I, J)** signaling pathways. TLR2, TLR4, and NLRP3 receptor genes **(K–M)** and protein **(N–Q)** expressions were detected by qRT-PCR and Western blot. ^ns^*P* > 0.05, ^*^P < 0.05; ^**^P < 0.01; ^***^P < 0.001.

The consequences indicated that, in contrast with those produced by way of SA113 *S. aureus*, Δ*lgt S. aureus* induced low levels of PGE_2_, IL-1β, IL-6, IL-10, and IL-8 secretion into neutrophil supernatants after infection of neutrophils for 9 h at MOI 3:1 and MOI 10:1 (*P* < 0.01, *P* < 0.001, Figures 1A–E). In MAPK and Caspase-1 pathways, the phosphorylation levels of ERK and P38 decreased in 15 min, 30 min, 60 min, and 120 min after infection with neutrophils by *S. aureus* Δ*lgt* compared with *S. aureus* SA113. Phosphorylation levels of Caspase-1 also decreased at 3 h, 6 h, and 9 h (Figures 1F–J). qRT-PCR results showed that compared with the SA113 *S. aureus*, the expression of TLR2, TLR4, and NLRP3 receptor genes in Δ*lgt S. aureus* was impaired at both MOI 3:1 and MOI 10:1, which was consistent with the results of the Western blotting (*P* < 0.001, Figures 1K–Q).

### COX-2 and mPGES-1 expressions in *S. aureus*-infected neutrophils and lipoproteins are essential

The precise functions of lipoproteins in the expression of COX-2 and mPGES-1 in neutrophils following *S. aureus* infection are still unknown. COX-2 and mPGES-1 mRNA levels were determined by qRT-PCR, and their protein levels were examined by Western blotting (extracts from *S. aureus*-infected neutrophils).

**Figure 2:**
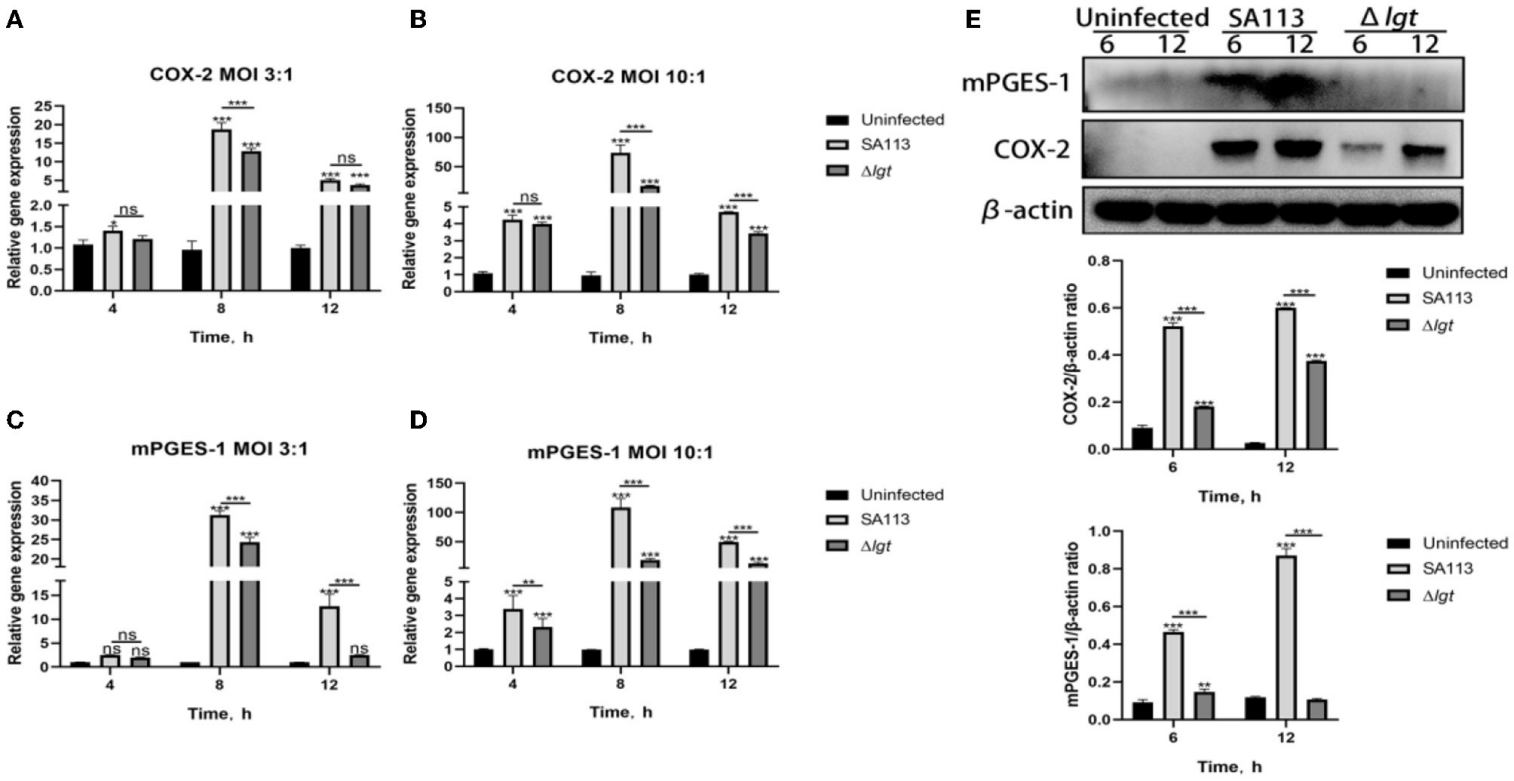
COX-2 and mPGES-1 expressions were induced by *S. aureus* lipoprotein. mRNA expression of COX-2 and mPGES-1 at 4 h, 8 h, and 12 h was detected by qRT-PCR [MOI 3:1 and MOI 10:1; **(A–D)**]. The protein expression of COX-2 and mPGES-1 was detected by Western blot at 6 h and 12 h [MOI 10:1, **(E)**]. ^ns^*P* > 0.05, ^*^P < 0.05; ^**^P < 0.01; ^***^P < 0.001.

The qRT-PCR results showed that in MOI 3:1, for COX-2 and mPGES-1, the SA113 *S. aureus* and Δ*lgt S. aureus* mRNA expression levels at 4 h did not differ significantly (*P*>0.05, Figures 2A, C). COX-2 is the same thing at 12 h, but at 8 h, there is substantial mRNA expression of COX-2 and mPGES-1 (*P* < 0.001, Figures 2A, C). Furthermore, mPGES-1 mRNA expression was the same at 12 h (*P* < 0.001, Figure 2C); in MOI 10:1, there was no discernible variation in COX-2 mRNA expression levels between SA113 *S. aureus* and Δ*lgt S. aureus* at 4 h but high COX-2 mRNA expression at 8 h and 12 h (*P* < 0.001, Figure 2B). In addition, mPGES-1 mRNA expression was higher at 4 h, 8 h, and 12 h (*P* < 0.01, *P* < 0.001, Figure 2D). The COX-2 and mPGES-1 protein expressions increased 6 h and 12 h after infection with SA113 *S. aureus* (MOI 10: 1) by the Western blot analysis (*P* < 0.001, Figure 2E). This was in contrast to that in cells infected with the Δ*lgt* strain or uninfected controls. These findings show that *S. aureus* lipoproteins are essential for inducing the expression of COX-2 and mPGES-1 in neutrophils.

### The secretion of PGE_2_ and cytokines mediated by neutrophils depends on TLR2, TLR4, and NLRP3 receptors

**Figure 3:**
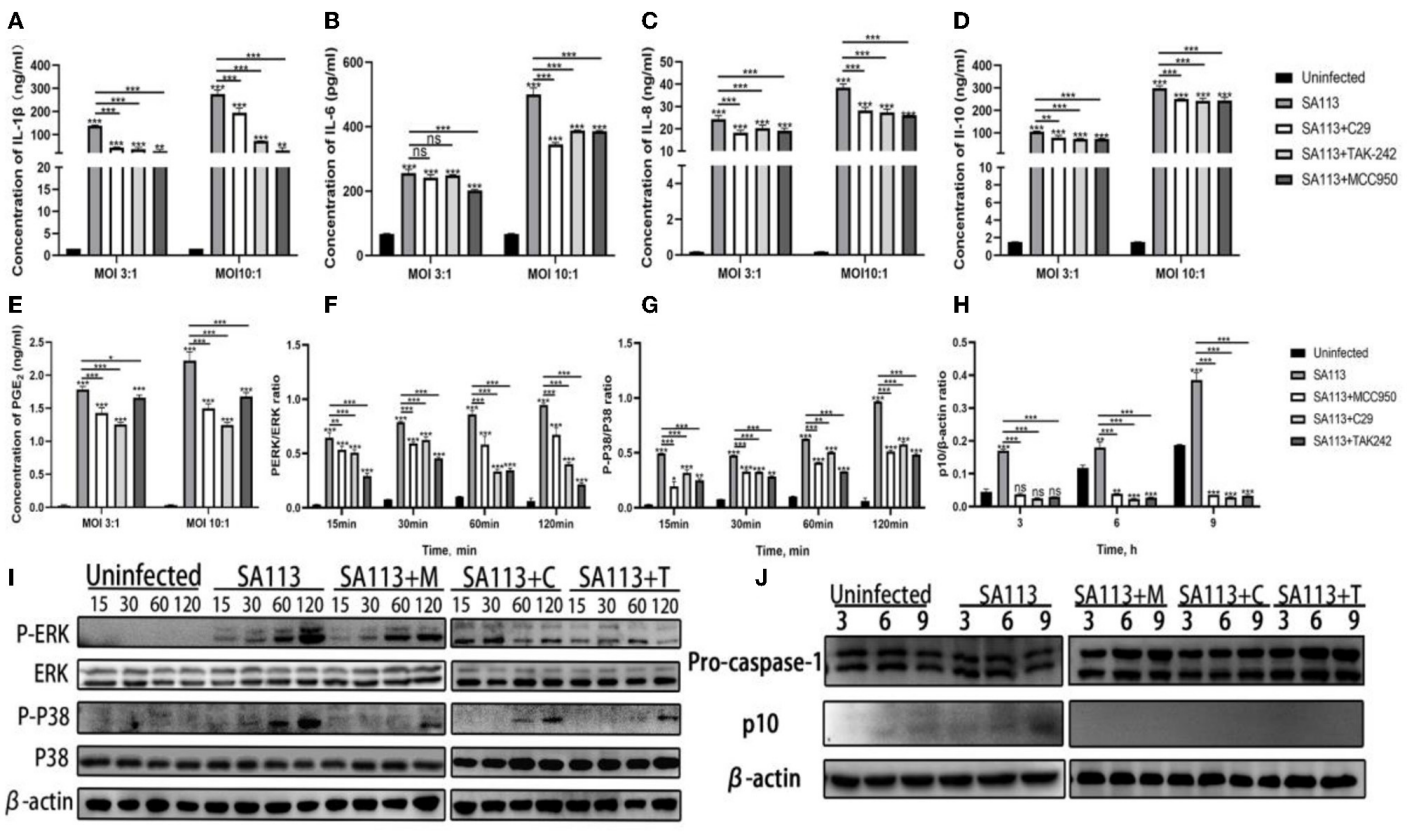
The secretion of PGE_2_ and cytokines mediated by neutrophils depends on TLR2, TLR4, and NLRP3 receptors. ELISA results showed the secretion levels of cytokines IL-1β **(A)**, IL-6 **(B)**, IL-8 **(C)**, IL-10 **(D)**, and PGE2 **(E)** in the SA113, C29, TAK-242, and MCC950 groups after neutrophils infection with MOI 3:1 and MOI 10:1. Western blot analysis revealed the activation of the MAPK **(F, G, I)** and Caspase-1 **(H, J)** signaling pathways. ^ns^*P* > 0.05, ^*^P < 0.05; ^**^P < 0.01; ^***^P < 0.001.

The above experiments indicated that *S. aureus* could not only activate the receptors of TLR2, TLR4, and NLRP3 in neutrophils but also induce the secretion of PGE_2_ and cytokines. Therefore, to better verify whether TLR2, TLR4, and NLRP3 receptors can affect the secretion of PGE_2_ and cytokines, we introduced TLR2 (C29), TLR4 (TAK-242), and NLRP3 (MCC950) receptor inhibitors for verification. ELISA results showed that compared with the C29, TAK-242, and MCC950 inhibitor groups, the SA113 group induced higher levels of PGE_2_, IL-1β, IL-10, IL-6, and IL-8 secretion at MOI 3:1 and MOI 10:1 (*P* < 0.001, Figures 3A–E) but except for IL-6 and PGE_2_ (Figures 3B, E) at MOI 3:1. At MOI 3:1, there was no discernible variation in IL-6 secretion in the SA113 group contrasted with the C29 and TAK-242 inhibitor groups (*P* > 0.05, Figure 3B). Western blot was used to detect the TLR2, TLR4, and NLRP3-mediated MAPK and Caspase-1 signaling pathways, and it was found that the inhibitor group was significantly inhibited contrasted with the SA113 group (*P* < 0.001, Figures 3F–J). These results demonstrate that the secretion of PGE_2_ and cytokines mediated by neutrophils depends on TLR2, TLR4, and NLRP3 receptors.

### TLR2, TLR4, and NLRP3 affect COX-2 and mPGES-1 expression in neutrophils after *S. aureus* infection

**Figure 4:**
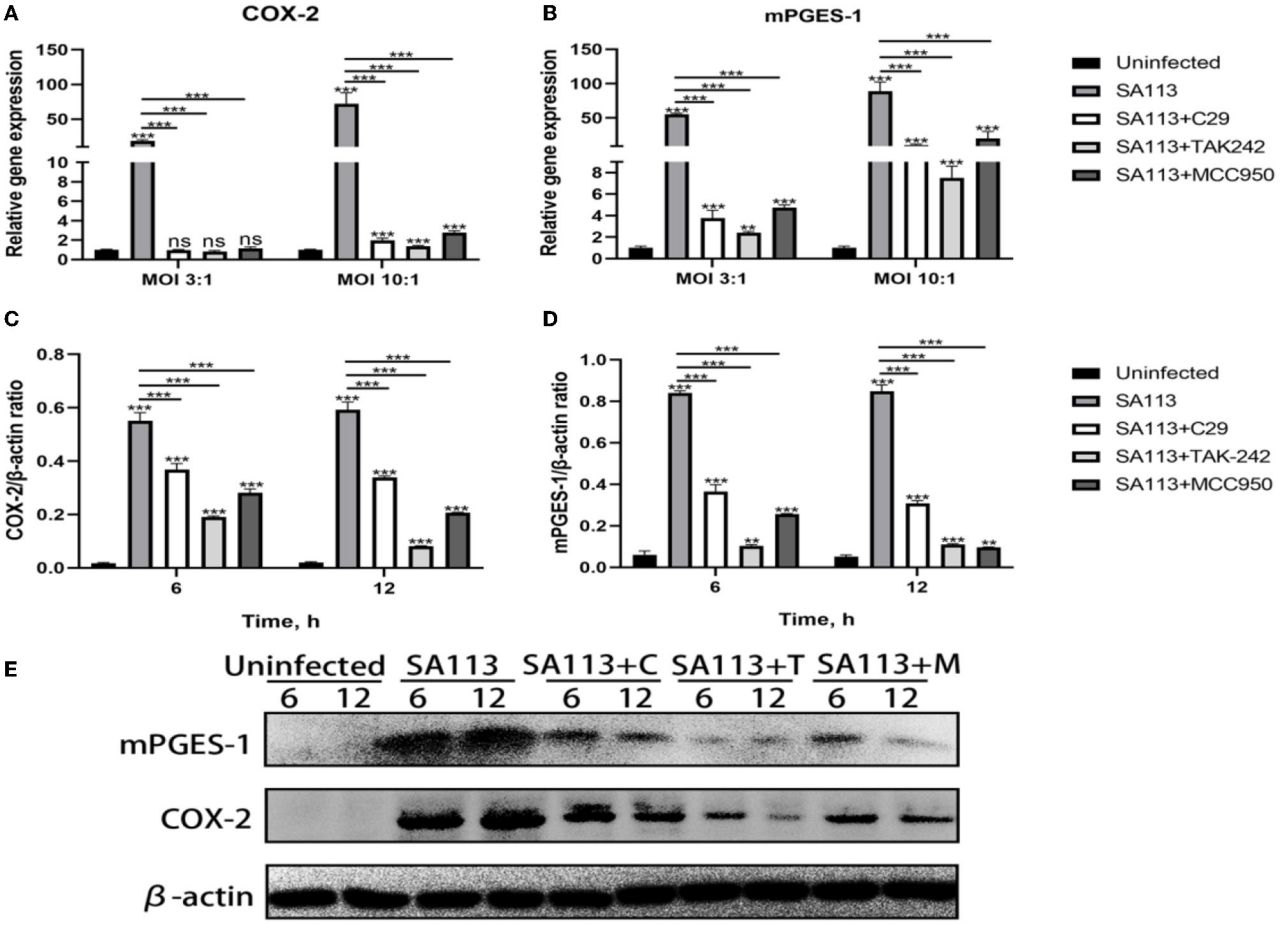
TLR2, TLR4, and NLRP3 affect COX-2 and mPGES-1 expressions in neutrophils after *S. aureus* infection. qRT-PCR results showed that C29, TAK-242, and MCC950 inhibitors at 8 h affected the expression of COX-2 and mPGES-1 in MOI 3:1 and MOI 10:1 **(A, B)**. Western blot results showed that C29, TAK-242, and MCC950 inhibitors at 6 h and 12 h affected the expression of COX-2 and mPGES-1 in MOI 10:1 **(C–E)**. ^ns^*P*>0.05, ^*^P < 0.05; ^**^P < 0.01; ^***^P < 0.001.

To verify the effect of TLR2, TLR4, and NLRP3 receptors on the COX-2, mPGES-1 gene, and protein, we pretreated neutrophils with C29, TAK-242, and MCC950 inhibitors and measured the expression of COX-2, mPGES-1 gene, and protein with qRT-PCR and Western blotting. qRT-PCR results showed that compared with the C29, TAK-242, and MCC950 inhibitor groups, the SA113 group induced higher level expression of COX-2 and the mPGES-1 at MOI 3:1 and MOI 10:1 (*P* < 0.001, Figures 4A, B). qRT-PCR and Western blot results were in agreement (*P* < 0.001, Figures 4C–E). These findings support the hypothesis that COX-2 and mPGES-1 expression in *S. aureus*-infected neutrophils is regulated by TLR2, TLR4, and the NLRP3 inflammasome.

### TLR2, TLR4, and NLRP3 receptors are involved in the intracellular killing of *S. aureus* by neutrophils, and *S. aureus* lacking lipoprotein is more likely to be killed, but PGE_2_ had no effect on cell killing

**Figure 5:**
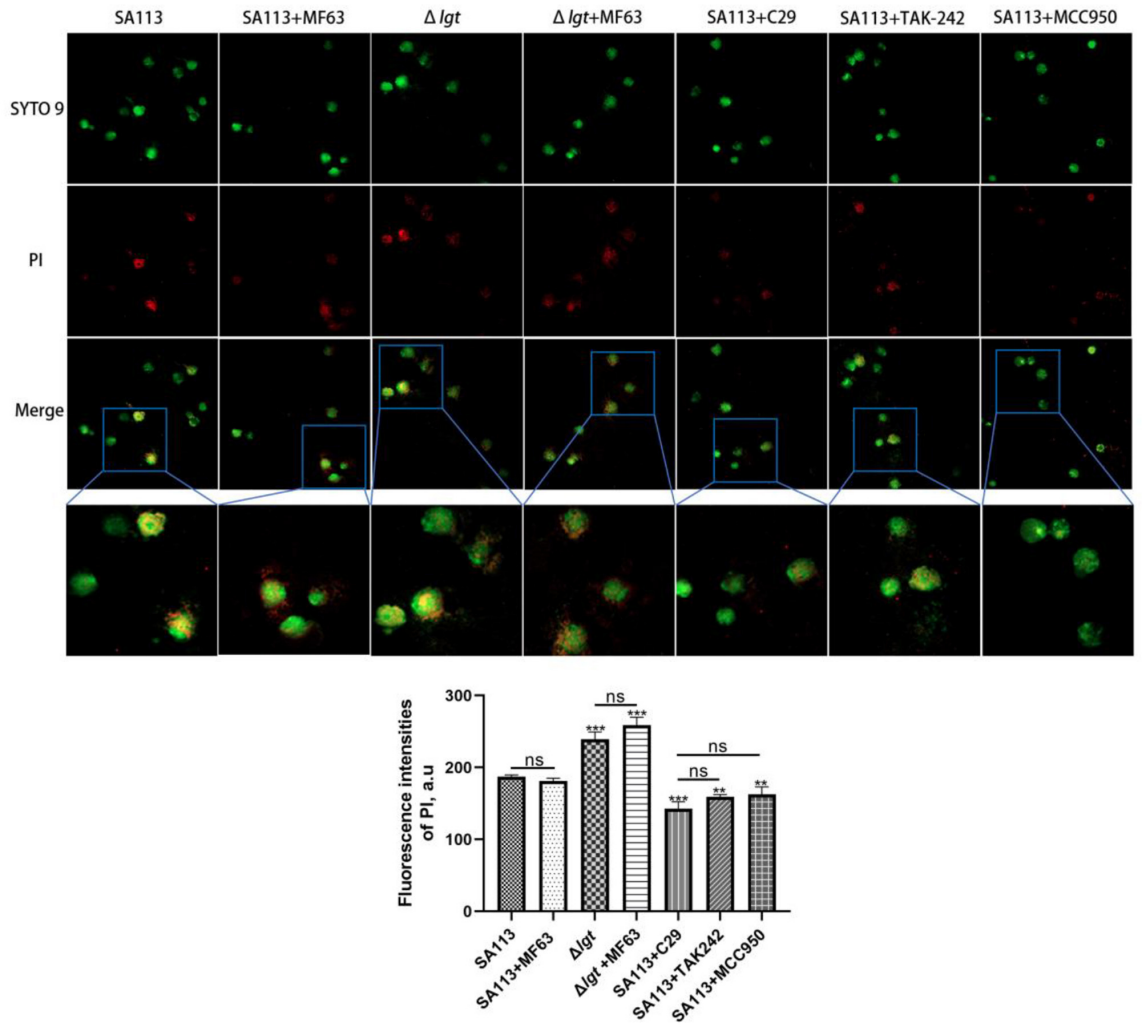
TLR2, TLR4, and NLRP3 receptors are involved in the intracellular killing of *S. aureus* by neutrophils, and *S. aureus* lacking lipoprotein is more likely to be killed, but PGE_2_ does not affect the killing of cells. Bacterial intracellular killing of PI (red)-labeled dead *S. aureus* within SYTO9-labeled neutrophils (green) was analyzed by microscopy assay (400×). ^ns^*P* > 0.05, ^*^P < 0.05; ^**^P < 0.01; ^***^P < 0.001.

In this study, we analyzed whether TLR2, TLR4, and NLRP3 receptors were involved in the killing ability of neutrophils against *S. aureus*, whether the lack of lipoprotein in *S. aureus* would affect this ability, and what effect PGE_2_ had on it. We introduced MF63, a PGE_2_ synthetase mPGES-1 inhibitor, to block the production of PGE_2_ in neutrophils after infection before testing the killing power. TLR2, TLR4, and NLRP3 inhibitors were also introduced to pretreat cells. In comparison to SA113 *S. aureus*, infection with Δ*lgt* resulted in low levels of bacterial survival in neutrophils (*P* < 0.001). The addition of the MF63 inhibitor group did not affect this process (*P* > 0.05). However, the number of *S. aureus* deaths decreased significantly in the C29 (*P* < 0.001), TAK-242 (*P* < 0.01), and MCC950 (*P* < 0.01) inhibitor groups compared with the SA113 group (Figure 5). These consequences reveal that inhibition of TLR2, TLR4, and NLRP3 receptors on neutrophils affected their killing ability against *S. aureus*, but PGE_2_ did not affect cell killing. Lipoprotein plays a vital role in the survival of *S. aureus*.

### Prostaglandin E_2_ can affect the secretion of pro-inflammatory factors, anti-inflammatory factors, and chemokines in neutrophils of cows infected with *S. aureus*

**Figure 6:**
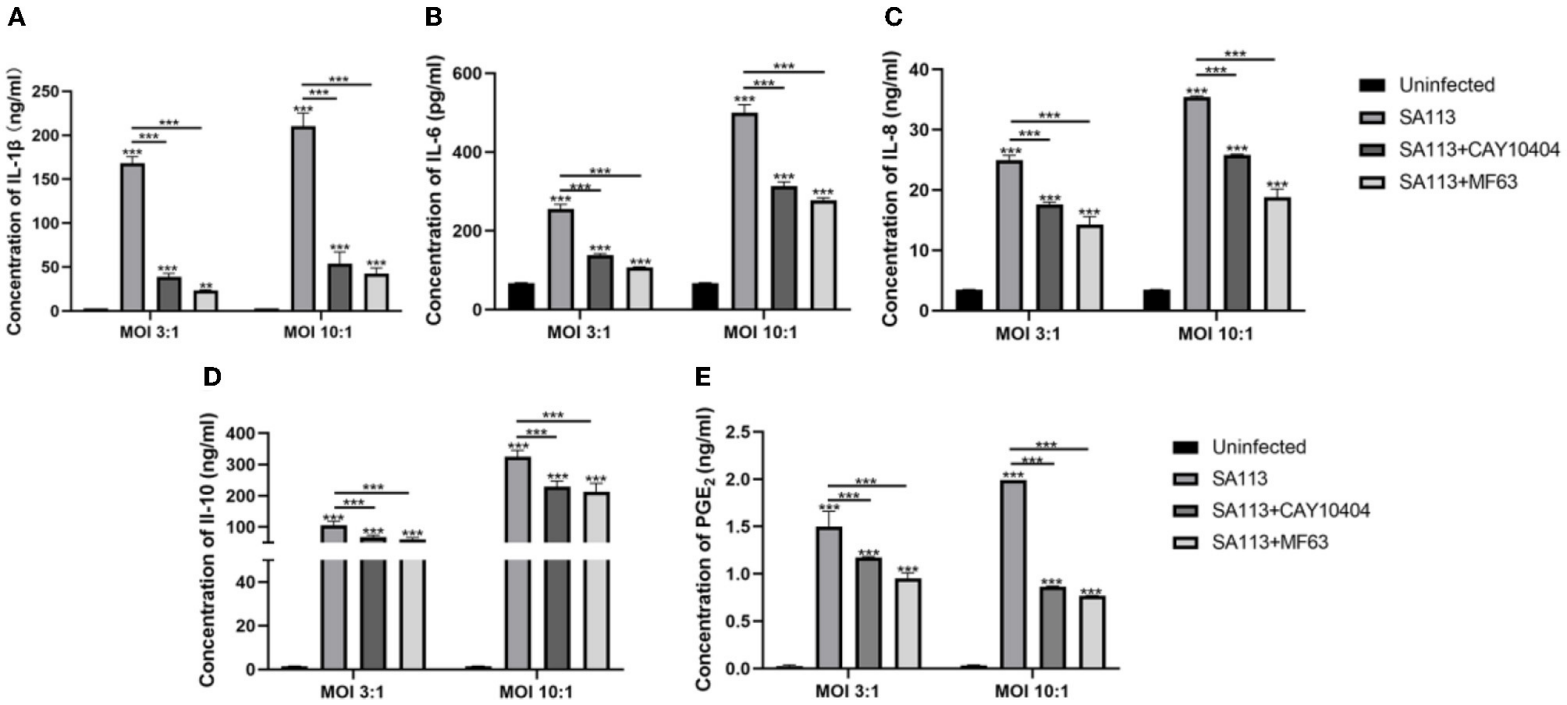
PGE_2_ can affect the secretion of pro-inflammatory factors, anti-inflammatory factors, and chemokines in neutrophils of cows infected with *S. aureus*. ELISA results showed that IL-1β **(A)**, IL-6 **(B)**, IL-8 **(C)**, IL-10 **(D)**, and PGE_2_ **(E)** were secreted in the SA113 group, the uninfected group, the CAY10404 group, and the MF63 group. ^ns^*P* > 0.05, ^*^P < 0.05; ^**^P < 0.01; ^***^P < 0.001.

To explore the role of PGE_2_ as an inflammatory mediator and other pro-inflammatory factors, anti-inflammatory factors, and chemokines, we introduced PGE_2_ synthetase COX-2 (CAY10404) and mPGES-1 (MF63) inhibitors to block the production of PGE_2_ in neutrophils after infection. ELISA results showed that the SA113 group induced higher levels of IL-1β, IL-6, IL-8, IL-10, and PGE_2_ secretion than the uninfected CAY10404 and MF63 groups (*P* < 0.001, Figures 6A–E).

## Discussion

*Staphylococcus aureus* (*S. aureus*) is a highly viable and widely distributed opportunistic pathogen (Mohammed et al., 2018). *S. aureus* causes economically valuable diseases in cows, including endometritis, vaginitis, and mastitis, which generate significant losses in the cow industry (Petit et al., 2010; Zhao et al., 2014; Shijin, 2022). Neutrophils are the first line of defense against bacterial pathogens that break through the epithelial barrier. Within minutes of bacterial invasion, neutrophils are drawn to the infection site in response to soluble substances such as chemokines and cytokines, where they ingest the pathogens and kill them (Guerra et al., 2017). PGE_2_ is one of the prostaglandins most closely associated with various inflammatory diseases (Pecchi et al., 2009) and induces inflammatory cytokines that mediate tissue harm at some stages in bacterial invasion (Tingting et al., 2018, 2019). Microbial membrane components of bacteria are generally identified by using TLRs, along with TLR2 and TLR4, which are activated through LPS and lipoproteins and are imperative factors for initiating innate immune responses such as inflammation (Langmann, 2015). NOD-like receptors also play a vital role in the infection of various pathogens and the process of intrinsic immunity, for example, NLRP3 (Singh and Jha, 2018). Therefore, we investigated whether PGE_2_ accumulation in neutrophils of *S. aureus*-infected cows is correlated with TLR2, TLR4, and NLRP3 receptors. As expected, we found that *S. aureus* promotes the secretion of PGE_2_ by activating TLR2, TLR4, and NLRP3 inflammasome signaling pathways in the neutrophils of cows. We also provide evidence that *S. aureus* is an important protein for its survival and activation of the inflammatory response.

Hayashi et al. (2010) found that TLR2 mediated the pro-inflammatory response to atherogenesis after *Porphyromonas gingivalis* infection, including the aggregation of macrophages and the release of inflammatory factors, for example, IFN-γ, IL-6, IL-1β, TNF-α, and IL-1β. Babelova et al. (2009) reported that dimeric glycans stimulate the mRNA expression of NLRP3 and pro-IL-1β through TLR2 and TLR4. Bauernfeind et al. (2009) found that the activation of TLR2, TLR3, TLR4, and TLR7 can activate the NLRP3 inflammasome in macrophages and Caspase-1. Therefore, we detected the expression of TLR2, TLR4, and NLRP3 receptor genes, proteins, and their associated signaling pathways, MAPK and Caspase-1, in dairy neutrophils infected by two strains of *S. aureus* SA113 and Δ*lgt*. And PGE_2_, IL-1β, IL-10, IL-6, and IL-8 secretion. We found that SA113 *S. aureus* induced higher levels of PGE_2_, IL-1β, IL-6, IL-10, and IL-8 secretion compared to neutrophils from cows infected with Δ*lgt S. aureus* or uninfected groups (Figures 1A–E). Furthermore, *S. aureus* SA113 induced higher levels of TLR2, TLR4, and NLRP3 receptor genes, and protein expression (Figures 1K–Q), and their mediated activation of MAPK, Caspase-1 signaling pathways (Figures 1F–J). The aforementioned findings indicate that the *S. aureus* lipoprotein component is one of the important components of neutrophil activation in *S. aureus*-infected cows. It induces not only the synthesis and secretion of neutrophils' PGE_2_, IL-1β, IL-10, IL-6, and IL-8 but also the expression of neutrophil TLR2, TLR4, and NLRP3 receptor genes and the activation of these three receptor-mediated related MAPK and Caspase-1 signaling pathways.

To verify the above experimental results, we added TLR2, TLR4, and NLRP3 receptor inhibitors to prove whether the secretion of PGE_2_, IL-6, IL-10, IL-1β, and IL-8 is mediated by TLR2, TLR4, and NLRP3 receptors. Impaired phosphorylation of MAPK and Caspase-1 signaling pathways indicated that TLR2, TLR4, and NLRP3 receptor inhibitors were effective (Figures 3F–J). Compared with the SA113 group, the C29, TAK-242, and MCC950 groups induced neutrophils to secrete PGE_2_, IL-6, IL-1β, IL-10, and IL-8 levels decreased (Figures 3A–E). It is suggested that TLR2, TLR4, and NLRP3 receptors on dairy neutrophils mediate the secretion of PGE_2_ and the secretion of cytokines IL-1β, IL-10, IL-6, and IL-8 after *S. aureus* infection of cow neutrophils. Our findings are consistent with those of previous studies (Stenzel et al., 2008; Müller et al., 2010).

Prostaglandin E_2_ is a recognized pro-inflammatory medium, mainly produced by COX-2 and mPGES-1 (Samuelsson et al., 2007). Here, we discovered that, compared to cells infected with *S. aureus* SA113, the *S. aureus* Δ*lgt* induced low levels of COX-2 and mPGES-1 expression in neutrophils (Figures 2A–E). mPGES-1 and COX-2 expressions were also impaired in C29, TAK-242, and MCC950 inhibitor group neutrophils (Figures 4A–E). These findings show that COX-2 and mPGES-1 expressions in *S. aureus*-infected neutrophils are dependent on *S. aureus* lipoprotein. In Addition, the presence of TLR2, TLR4, and NLRP3 in neutrophils was crucial for *S. aureus*-induced COX-2 and mPGES-1 expressions. This result is consistent with the previous research of Jindi et al. in mouse peritoneal macrophages (Wu et al., 2020).

*Staphylococcus aureus* is a highly adaptive gram-positive pathogen (Gorwitz et al., 2008). It can cause serious illnesses such as pneumonia, endocarditis, and bacteremia by infecting different organs. The innate immunity of the human body sends out expert phagocytic cells in response to bacterial infection, such as neutrophils and macrophages, to engulf the invading pathogens and kill them (Thammavongsa et al., 2015). The literature reports that *S. aureus* can survive and proliferate in specialized phagocytes such as neutrophils, macrophages, and monocytes, and this phenomenon may be one of the reasons for the spread of *S. aureus* in the host (Horn et al., 2017). In this study, we examined the killing function of neutrophils against *S. aureus*. The result found that compared with *S. aureus* SA113 infected cells, *S. aureus* Δ*lgt* is more easily killed by neutrophils. To determine whether PGE_2_ and TLR2, TLR4, and NLRP3 receptors affect this effect, we introduced MF63, the main synthetase mPGES-1 inhibitor of PGE_2_, and TLR2 (C29), TLR4 (TAK-242), and NLRP3 (MCC950) receptor inhibitors to observe the killing effect of neutrophils on *S. aureus*. We found that the addition of the MF63 group had no significant effect on neutrophil-killing of *S. aureus*, but the addition of C29, TAK-242, and MCC950 significantly inhibited this effect. We also found that *S. aureus* Δ*lgt* had decreased survival in neutrophils compared to *S. aureus* SA113 (Figure 5). These findings advise that PGE_2_ no longer has an effect on bacterial intracellular killing, and *S. aureus* lipoprotein plays a role in cell survival.

Neutrophils can regulate the immune response through the production of the cytokines IL-12 and IL-10 and the lipid mediators PGE_2_ and LTB4, as reported by Balderramas et al. This regulation may be in a pro-inflammatory or anti-inflammatory mode (Balderramas et al., 2014). PGE_2_ has been reported to play a key role in human endometrial repair by regulating the expression of connective tissue growth factor and IL-8 (Maybin et al., 2011). It has been shown that PGE_2_ induces IL-6 production (Subedi et al., 2017). IL-1β promotes RA synovial infiltration and increases PGE_2_ release, which, in turn, promotes IL-1β synthesis (Nilsson et al., 2017). IL-10 reduces the secretion of PGE_2_ in osteoarthritic synovial fibroblasts and has a protective effect on articular cartilage (Botha-Scheepers et al., 2008; Järvinen et al., 2008). PGE_2_ also inhibits dendritic cell and macrophage maturation and antigen presentation via EP2/EP4 receptors and increases IL-10 production while decreasing IL-12, TNF-, and IL-1 production (Harizi et al., 2001). So is there a link between PGE_2_ and these cytokines in *S. aureus*-infected neutrophils? The above reports suggest a possible synergistic or antagonistic relationship between PGE_2_ and IL-6, IL-8, IL-1β, and IL-10. Therefore, we added MF63, an inhibitor of mPGES-1, and CAY10404, an inhibitor of COX-2, to neutrophils from *S. aureus*-infected cows to investigate whether blocking the secretory pathway of PGE_2_ would lead to changes in the secretion of IL-6, IL-10, IL-1β, and IL-8. The results found that the secretion of PGE_2_ (Figure 6E) and IL-1 (Figure 6A), IL-6 (Figure 6B), IL-8 (Figure 6C), and IL-10 (Figure 6D) cytokines were restricted when MF63 inhibitor and CAY10404 inhibitor were added, suggesting that there may be a synergistic relationship between PGE_2_ and IL-1β, IL-8, IL-6, and IL-10 in neutrophils of *S. aureus*-infected cows and that they jointly exert immunomodulatory effects.

We demonstrate an association between PGE_2_ and PRR-mediated innate immune responses of cow neutrophils to *S. aureus*. TLR2, TLR4, and NLRP3 receptors are involved in the synthesis and secretion of PGE_2_ in *S. aureus*-infected cow neutrophils. In this process, we unexpectedly found that *S. aureus* lipoprotein is an important protein in activating the inflammatory response and the survival of *S. aureus*. Nevertheless, it is still uncertain whether PGE_2_ and its analogs can be utilized as a medication or an immunomodulator to guard against infectious disorders brought on by *S. aureus*. Therefore, more research is required, particularly medical testing.

## Data availability statement

The original contributions presented in the study are included in the article/supplementary material, further inquiries can be directed to the corresponding author.

## Ethics statement

All animal experiments were performed according to the regulations of the Administration of Affairs Concerning Experimental Animals in China. The experimental protocol was approved by the Animal Welfare and Research Ethics Committee of the Inner Mongolia Agricultural University (Approval ID: NND202103).

## Author contributions

YQ, XJ, YJ, WM, BL, SZ, YS, and JC: preparation, creation, and presentation of the published study, specifically writing the initial draft (including substantive translation). KZ: methodology, data analysis, and original draft. All authors contributed to the article and approved the submitted version.

## References

[B1] Amulic B. Cazalet C. Hayes G. L. Metzler K. D. Zychlinsky A. (2012). Neutrophil function: from mechanisms to disease. Annu. Rev. Immunol. 30, 459. 10.1146/annurev-immunol-020711-074942 22224774

[B2] Babelova A. Moreth K. Tsalastra-Greul W. Zeng-Brouwers J. Eickelberg O. Young M. F.. (2009). Biglycan, a danger signal that activates the NLRP3 inflammasome via toll-like and P2X receptors. J. Biol. Chem. 284, 24035–24048. 10.1074/jbc.M109.014266 19605353 PMC2781998

[B3] Balderramas H. A. Penitenti M. Rodrigues D. R. Bachiega T. F. Fernandes R. K. Ikoma M. R. V.. (2014). Human neutrophils produce IL-12, IL-10, PGE2 and LTB4 in response to *Paracoccidioides brasiliensis*. involvement of TLR2, mannose receptor and dectin-1. Cytokine 67, 36–43. 10.1016/j.cyto.2014.02.004 24680480

[B4] Bauernfeind F. G. Horvath G. Stutz A. Alnemri E. S. MacDonald K. Speert D.. (2009). Cutting Edge: NF-κB activating pattern recognition and cytokine receptors license NLRP3 inflammasome activation by regulating NLRP3 expression. J. Immunol. 183, 787–791. 10.4049/jimmunol.0901363 19570822 PMC2824855

[B5] Botha-Scheepers S. Watt I. Slagboom E. de Craen A. J. M. Meulenbelt I. Rosendaal F. R.. (2008). Innate production of tumour necrosis factor alpha and interleukin 10 is associated with radiological progression of knee osteoarthritis. Ann. Rheum. Dis. 67, 1165. 10.1136/ard.2007.084657 18029383

[B6] Catley M. C. Chivers J. E. Cambridge L. M. Holden N. Slater D. M. Staples K. J.. (2003). IL-1β-dependent activation of NF-κB mediates PGE2 release via the expression of cyclooxygenase-2 and microsomal prostaglandin E synthase. FEBS Lett. 547, 75–79. 10.1016/S0014-5793(03)00672-0 12860389

[B7] Ciliberti M. G. Albenzio M. Claps S. Santillo A. Marino R. Caroprese M. (2021). NETosis of peripheral neutrophils isolated from dairy cows fed olive pomace. Front. Vet. Sci. 8, 626314. 10.3389/fvets.2021.626314 33996961 PMC8118642

[B8] Dsa B. C. Bgs B. J. D. M. Bentley R. E. Martin A. Y. Jones O. (2022). Mitochondria in human neutrophils mediate killing of *Staphylococcus aureus*. Redox Biol. 9, 102225. 10.1016/j.redox.2021.102225 34959099 PMC8758915

[B9] Gorwitz R. J. Kruszon-Moran D. McAllister S. K. McQuillan G. McDougal L. K. Fosheim G. E.. (2008). Changes in the prevalence of nasal colonization with *Staphylococcus aureus* in the United States. J. Infect. Dis. 197, 1226–1234. 10.1086/533494 18422434

[B10] Guerra F. E. Borgogna T. R. Patel D. M. Sward E. W. Voyich J. M. (2017). Epic immune battles of history: neutrophils vs. Staphylococcus aureus. Front. Cell. Infect. Microbiol. 7, 286. 10.3389/fcimb.2017.00286 28713774 PMC5491559

[B11] Harizi H. Juzan M. Grosset C. Rashedi M. Gualde N. (2001). Dendritic cells issued *in vitro* from bone marrow produce PGE2 that contributes to the immunomodulation induced by antigen-presenting cells. Cell. Immunol. 209, 19–28. 10.1006/cimm.2001.1785 11414733

[B12] Hashimoto M. Tawaratsumida K. Kariya H. Aoyama K. Tamura T. Suda Y. (2006a). Lipoprotein is a predominant Toll-like receptor 2 ligand in *Staphylococcus aureus* cell wall components. Int. Immunol. 18, 355–362. 10.1093/intimm/dxh374 16373361

[B13] Hashimoto M. Tawaratsumida K. Kariya H. Kiyohara A. Suda Y. Krikae F.. (2006b). Not lipoteichoic acid but lipoproteins appear to be the dominant immunobiologically active compounds in *Staphylococcus aureus*. J. Immunol. 177, 3162–3169. 10.4049/jimmunol.177.5.3162 16920954

[B14] Hayashi C. Madrigal A. G. Liu X. Ukai T. Goswami S. Gudino C. V.. (2010). *P*athogen-mediated inflammatory atherosclerosis is mediated in part via Toll-like receptor 2-induced inflammatory responses. J. Innate Immun. 2, 334–343. 10.1159/000314686 20505314 PMC2895755

[B15] Horn J. Stelzner K. Rudel T. Fraunholz M. (2017). Inside job: *Staphylococcus aureus* host-pathogen interactions. Int. J. Med. Microbiol. 2017, S1438422117303168. 10.1016/j.ijmm.2017.11.009 29217333

[B16] Järvinen K. Vuolteenaho K. Nieminen R. Moilanen T. Knowles R. G. Moilanen E. (2008). *S*elective iNOS inhibitor 1400W enhances anti-catabolic IL-10 and reduces destructive MMP-10 in OA cartilage. Survey of the effects of 1400W on inflammatory mediators produced by OA cartilage as detected by protein antibody array. Clin. Exp. Rheumatol. 26, 275. 18565249

[B17] Kessel K. P. M. V. Bestebroer J. Strijp J. A. G. V. (2014). Neutrophil-Mediated Phagocytosis of *Staphylococcus aureus*. Front. Immunol. 5, 467. 10.3389/fimmu.2014.00467 25309547 PMC4176147

[B18] Kevin M. R. DeLeo F. R. (2012). Neutrophils in innate host defense against *Staphylococcus aureus* infections. Semin. Immunopathol. 34, 237–259. 10.1007/s00281-011-0295-3 22080185 PMC3271231

[B19] Langmann T. (2015). Intracellular toll-like receptors help retinal microglia sense corneal infections. Invest. Ophthalmol. Vis. Sci. 56, 7387. 10.1167/iovs.15-18268 26574797

[B20] Maybin J. A. Hirani N. Jabbour H. N. Critchley H. O. D. (2011). Novel roles for hypoxia and prostaglandin E2 in the regulation of IL-8 during endometrial repair. Am. J. Pathol. 178, 1245–1256. 10.1016/j.ajpath.2010.11.070 21356375 PMC3047791

[B21] Mohammed Y. H. E. Manukumar H. M. Rakesh K. P. Karthik C. S. Mallu P. Qin H.-L. (2018). Vision for medicine: *Staphylococcus aureus* biofilm war and unlocking key's for anti-biofilm drug development. Microb. Pathog. 123, 339–347. 10.1016/j.micpath.2018.07.002 30057355

[B22] Müller P. Müller-Anstett M. Wagener J. Gao Q. Kaesler S. Schaller M.. (2010). The *Staphylococcus aureus* lipoprotein SitC colocalizes with Toll-like receptor 2 (TLR2) in murine keratinocytes and elicits intracellular TLR2 accumulation. Infect. Immun. 78, 4243–4250. 10.1128/IAI.00538-10 20679445 PMC2950364

[B23] Nakanishi M. Montrose D. C. Clark P. Nambiar P. R. Belinsky G. S. Claffey K. P.. (2008). Genetic deletion of mPGES-1 suppresses intestinal tumorigenesis. Cancer Res. 68, 3251–3259. 10.1158/0008-5472.CAN-07-6100 18451151

[B24] Nilsson A. Elander L. Hallbeck M. Kugelberg U. Ö. Engblom D. Blomqvist A. (2017). The involvement of prostaglandin E2 in interleukin-1β evoked anorexia is strain dependent. Brain Behav. Immunity 60, 27–31. 10.1016/j.bbi.2016.06.014 27375005

[B25] Pecchi E. Dallaporta M. Jean A. Thirion S. Troadec J.-D. (2009). Prostaglandins and sickness behavior: old story, new insights. Physiol. Behav. 97, 279–292. 10.1016/j.physbeh.2009.02.040 19275907

[B26] Petit T. Spergser J. Rosengarten R. Aurich J. (2010). Prevalence of potentially pathogenic bacteria as genital pathogens in dairy cattle. Reproduct. Domestic Anim. 44, 88–91. 10.1111/j.1439-0531.2007.01002.x 18537907

[B27] Prince L. R. Whyte M. K. Sabroe I. Parker L. C. (2011). The role of TLRs in neutrophil activation. Curr. Opin. Pharmacol. 11, 397–403. 10.1016/j.coph.2011.06.007 21741310

[B28] Rodríguez-Yáñez M. Brea D. Arias S. Blanco M. Pumar J. M. Castillo J.. (2012). Increased expression of Toll-like receptors 2 and 4 is associated with poor outcome in intracerebral hemorrhage. J. Neuroimmunol. 247, 75–80. 10.1016/j.jneuroim.2012.03.019 22498099

[B29] Samuelsson B. Morgenstern R. Jakobsson P. J. (2007). Membrane prostaglandin e synthase-1: a novel therapeutic target. Pharmacol. Rev. 59, 207–224. 10.1124/pr.59.3.1 17878511

[B30] Sheldon I. M. Cronin J. G. Healey G. D. Gabler C. Heuwieser W. Streyl D.. (2014). Innate immunity and inflammation of the bovine female reproductive tract in health and disease. Reproduct. Domest. Animals 148, R41–51. 10.1530/REP-14-0163 24890752

[B31] Shijin G. (2022). Effect of chinese herbal medicine on pathogenic bacteria of cow mastitis. Animal Husbandry Feed Sci. 12, 3.

[B32] Singh S. Jha S. (2018). NLRs as helpline in the brain: mechanisms and therapeutic implications. Mol. Neurobiol. 55, 8154–8178. 10.1007/s12035-018-0957-4 29508284

[B33] Stenzel W. Soltek S. Sanchez-Ruiz M. Akira S. Miletic H. Schlüter D.. (2008). Both TLR2 and TLR4 are required for the effective immune response in *Staphylococcus aureus*-induced experimental murine brain abscess. Am. J. Pathol. 172, 132–145. 10.2353/ajpath.2008.070567 18165267 PMC2189630

[B34] Stoll H. Dengjel J. Nerz C. Götz F. (2005). *Staphylococcus aureus* deficient in lipidation of prelipoproteins is attenuated in growth and immune activation. Infect. Immun. 73, 2411–2423. 10.1128/IAI.73.4.2411-2423.2005 15784587 PMC1087423

[B35] Subedi L. Venkatesan R. Sun Y. K. (2017). Neuroprotective and anti-inflammatory activities of allyl isothiocyanate through attenuation of JNK/NF-κB/TNF-α signaling. Int. J. Mol. Sci. 18, 1423. 10.3390/ijms18071423 28671636 PMC5535914

[B36] Thammavongsa V. Kim H. K. Missiakas D. Schneewind O. (2015). Staphylococcal manipulation of host immune responses. Nat. Rev. Microbiol 13, 529–543. 10.1038/nrmicro3521 26272408 PMC4625792

[B37] Tingting L. Liu B. Guan H. Mao W. Wang L. Zhang C.. (2018). PGE_2_ increases inflammatory damage in Escherichia coli-infected bovine endometrial tissue in vitro via the EP4-PKA signaling pathway. Biol. Reprod. 100, 175–186. 10.1093/biolre/ioy162 30010723

[B38] Tingting L. Mao W. Liu B. Gao R. Zhang S. Wu J. (2019). LP induced/mediated PGE2 synthesis through activation of the ERK/NF-κB pathway contributes to inflammatory damage triggered by Escherichia coli-infection in bovine endometrial tissue. Vet. Microbiol. 232, 96–104. 10.1016/j.vetmic.2019.03.005 31030852

[B39] Wan M. Tang X. Rekha R. S. Muvva S. S. V. J. R. Brighenti S. Agerberth B.. (2018). Prostaglandin E-2 suppresses hCAP18/LL-37 expression in human macrophages via EP2/EP4: implications for treatment of *Mycobacterium tuberculosis* infection. FASEB J. 32, 2827–2840. 10.1096/fj.201701308 29401596

[B40] Wu J. Liu B. Mao W. Feng S. Yao Y. Bai F.. (2020). Prostaglandin E2 regulates activation of mouse peritoneal macrophages by *Staphylococcus aureus* through toll-like receptor 2, toll-like receptor 4, and NLRP3 inflammasome signaling. J. Innate Immun. 12, 604. 10.1159/000499604 31141808 PMC7098297

[B41] Zhao J.-L. Ding Y.-X. Zhao H.-X. He X.-L. Li P.-F. Li Z.-F.. (2014). Presence of superantigen genes and antimicrobial resistance in *Staphylococcus isolates* obtained from the uteri of dairy cows with clinical endometritis. Vet. Rec. 175, 352–352. 10.1136/vr.102302 24989035

